# 3D MR Thermometry Using Bi‐Directional Segmented EPI for Transcranial‐Focused Ultrasound

**DOI:** 10.1002/mrm.70195

**Published:** 2025-11-23

**Authors:** Michael Malmberg, Seong‐Eun Kim, John Roberts, Lubdha Shah, Dennis L. Parker, Henrik Odéen

**Affiliations:** ^1^ Department of Biomedical Engineering University of Utah Salt Lake City Utah USA; ^2^ Department of Radiology and Imaging Sciences University of Utah Salt Lake City Utah USA

**Keywords:** 3D, EPI, focused ultrasound, MRgFUS, segmented EPI, temperature, thermometry

## Abstract

**Purpose:**

To evaluate the feasibility of replacing clinically utilized 2D thermometry with 3D segmented EPI‐based thermometry with equivalent accuracy, precision, and scan time.

**Methods:**

A 3D segmented EPI (segEPI) trajectory was modified to allow readouts along both the forward and reverse direction for each phase encoding line, and the sequence was integrated into the existing clinical protocol. Focused ultrasound sonications were performed in a tissue‐mimicking gelatin phantom with the clinical standard scan and the proposed scan across multiple repetitions. Accuracy, precision and extent of the measured heating was compared at varied levels of zero‐filled interpolation. Non‐heating in vivo brain scans were performed to evaluate phase drift and precision improvements from echo combination.

**Results:**

A 3D segEPI thermometry was found to have high agreement with 2D clinical thermometry while providing greater knowledge of the extent of heating. Temperature limits of agreement remained below ±1°C for both the uncombined and combined‐echo cases when zero‐filled interpolation was performed to 0.5 mm in‐plane voxel spacing or smaller. The full‐width at half maximum of the heating distribution was found to be slightly higher (∼17%) for 3D segEPI, potentially due to uncorrected heating‐induced chemical shift. Echo combination in segEPI is found to improve precision of temperature estimates.

**Conclusions:**

A 3D segEPI thermometry is shown to be a promising alternative to clinical 2D thermometry, with possibility of rapid integration into existing clinical workflows.

## 1 Introduction

High‐intensity focused ultrasound (HIFU) ablation is a noninvasive surgical procedure capable of targeting and destroying deep tissue regions with millimeter accuracy. These procedures necessitate highly accurate and precise MR temperature imaging (MRTI) to monitor the procedure in real time and to evaluate treatment endpoints. Typically, desirable temperature accuracy and precision is within approximately ±1°C [1] and the measurements should have sufficient temporal resolution to track the dynamic heating, which generally corresponds to an image frame every 3–5 s. MR‐guided transcranial HIFU is currently regulatory approved to ablate the thalamic ventral intermediate nucleus (VIM) for treatment of essential and Parkinsonian tremor [2, 3], but other applications, such as in oncology, have been suggested in the literature or are in clinical trials [4, 5].

Current clinical MRTI for transcranial applications perform single slice, 2D proton resonance frequency shift (PRFS) thermometry [6], in which phase images between two time points are subtracted and used to deduce a local temperature‐induced resonance frequency change which can be translated into a temperature change [7]. The limited k‐space data needed to be acquired for the single slice measurements can be acquired with sufficiently high spatial and temporal resolution, but 2D scanning limits the ability to accurately localize the focal spot position in three dimensional space and to assess the full extent of HIFU heating throughout the 3D volume. Instead, during the targeting and verification steps of the procedure, multiple separate sonications are performed and monitored using orthogonal planes (i.e., sagittal, coronal and axial). The focal position is adjusted until accurate targeting is confirmed. This results in multiple sonications and acquisitions, typically at least one in each orientation, with potential adjustments to the phase encoding direction for each slice. This enables accurate targeting in all three spatial directions, but results in longer procedure times. During the treatment, a single slice through the predetermined focal location is imaged repeatedly during HIFU sonication [8] until the MRTI shows that ablation temperatures for the target region have been reached (typical size of the immediate post‐HIFU lesion is ∼6 × 5 × 7 mm). Throughout the procedure, the imaging orientation is often cycled to monitor the extent of the heating in all directions, but with a single 2D slice, it is not possible to assess the heat distribution throughout the targeted volume in a single scan. Since the focal spot is often angled in a single or double oblique direction relative to the three orthogonal imaging planes, it is further possible that heating is occurring outside the imaged slices, which could potentially damage adjacent structures and cause side effects. Lastly, 2D MRTI typically employs a slice thickness comparable to the focal spot dimension in this direction, which can lead to partial volume inaccuracies in the produced MRTI maps, as 2D imaging can only interpolate the data in the two in‐plane directions [9]. These limitations could potentially be overcome by using fully volumetric 3D MRTI to monitor the procedures. With 3D MRTI, the full spatial extent of the oblique focal spot would be imaged in every acquisition. Since the data is 3D, it could be interpolated in all directions to minimize partial volume effects for more accurate measurements, especially of the peak temperatures, which are known to be sensitive to spatial resolution [10].

Much work has been done to develop fast 3D trajectories for PRFS thermometry. However, many of these approaches rely on complicated and vendor‐dependent acquisition approaches such as non‐Cartesian radial and spiral acquisition schemes [11, 12, 13, 14], which, in addition to their more limited availability, require additional, more time‐consuming reconstruction techniques. Using different pulse sequences across MRI vendors makes it harder for HIFU system vendors to acquire, process and visualize the data and to validate the approach for regulatory approval. Other approaches in the literature use more advanced reconstruction methods to reconstruct undersampled data [15, 16, 17], such as constrained or model‐based approaches. These approaches are either computationally time‐consuming or rely on tissue‐specific acoustic and/or thermal properties, which has hindered their translation. As a result, none of these approaches for volumetric MRTI have yet been integrated into the clinical workflow for focused ultrasound applications. For clinical translation of 3D thermometry to advance in transcranial applications, a 3D sequence must be developed that can be easily integrated into existing workflows while providing equivalent clinical information.

In this work, we investigate the feasibility of MRTI using a 3D segmented GRE EPI (segEPI) sequence, which is readily available on most major MRI vendors' platforms. To maximize sampling efficiency for improved SNR, we implemented bidirectional sampling on the oscillating readout gradient. The performance of the bidirectional 3D segmented EPI MRTI sequence was compared to the clinically utilized single‐slice 2D multi‐echo spoiled GRE sequence, hereafter referred to using the vendor's (Insightec, Tirat‐Carmel, Israel) acronym multi‐echo‐multi‐plane (MEMP) in conjunction with heating on a clinical MR‐guided transcranial HIFU system (Exablate, Insightec). Since the acquisition scheme is Cartesian, the reconstruction is fast, computationally efficient, does not require any a priori information, and can be performed using the same reconstruction pipeline as the currently used clinical sequence. We investigate the comparative accuracy and precision of MRTI using these two sequences with the clinical HIFU system, with the aim to demonstrate the feasibility of achieving 3D MRTI that covers the full focal spot with accuracy, precision and scan time comparable to the currently‐used clinical 2D approach. By utilizing 3D MRTI, transcranial HIFU treatments could become potentially more efficient by reducing the number of pre‐treatment targeting and calibration sonications and safer by accurately monitoring the full extent of the oblique focal spot in every sonication.

## 2 Methods

All experiments were performed on a clinically available transcranial HIFU system (Exablate, Insightec) connected to a 3 T 70‐cm wide bore MRI scanner (Skyra, Siemens Healthineers, Erlangen, Germany). All data was acquired using a dual RF receive coil setup (Insightec) consisting of two loop coils embedded into the membrane containing the water bolus of the HIFU system [18]. Free field hydrophone measurements (HNR‐0400, Onda, Sunnyvale CA) of the transducer used in this study resulted in pressure FWHM of 1.4 mm × 1.4 mm × 2.8 mm. In the evaluation of the feasibility of replacing the 2D clinical MEMP sequence with a 3D segEPI sequence, the following measures were deemed important to investigate: (1) accuracy and precision of the measured temperatures over time; (2) size or extent of the focal heating; (3) accuracy of the measured location of focal heating; and (4) temporal stability and temporal resolution of the PRF temperature maps. Phantom (with HIFU heating) and in vivo (without HIFU heating) experiments were performed to evaluate each of these points, with five repeated sonications performed for ex vivo phantom experiments and ∼2 min of scanning performed for in vivo volunteer scans.

The single slice 2D MEMP scan (a clinically approved protocol) is a 5 contrast (echo) spoiled GRE scan with the following parameters: TR = 27.15 ms, TE = [3.12, 7.94, 12.76, 17.58, 22.40] ms, bipolar imaging readout, FOV = [280 × 280 × 3] mm^3^, acquired matrix size = [256 × 128], spatial resolution = [1.09 × 2.19 × 3] mm^3^, flip angle = 12°, frequency encoding bandwidth = 279 Hz/pixel, time per image frame ≈3.48 s.

**FIGURE 1:**
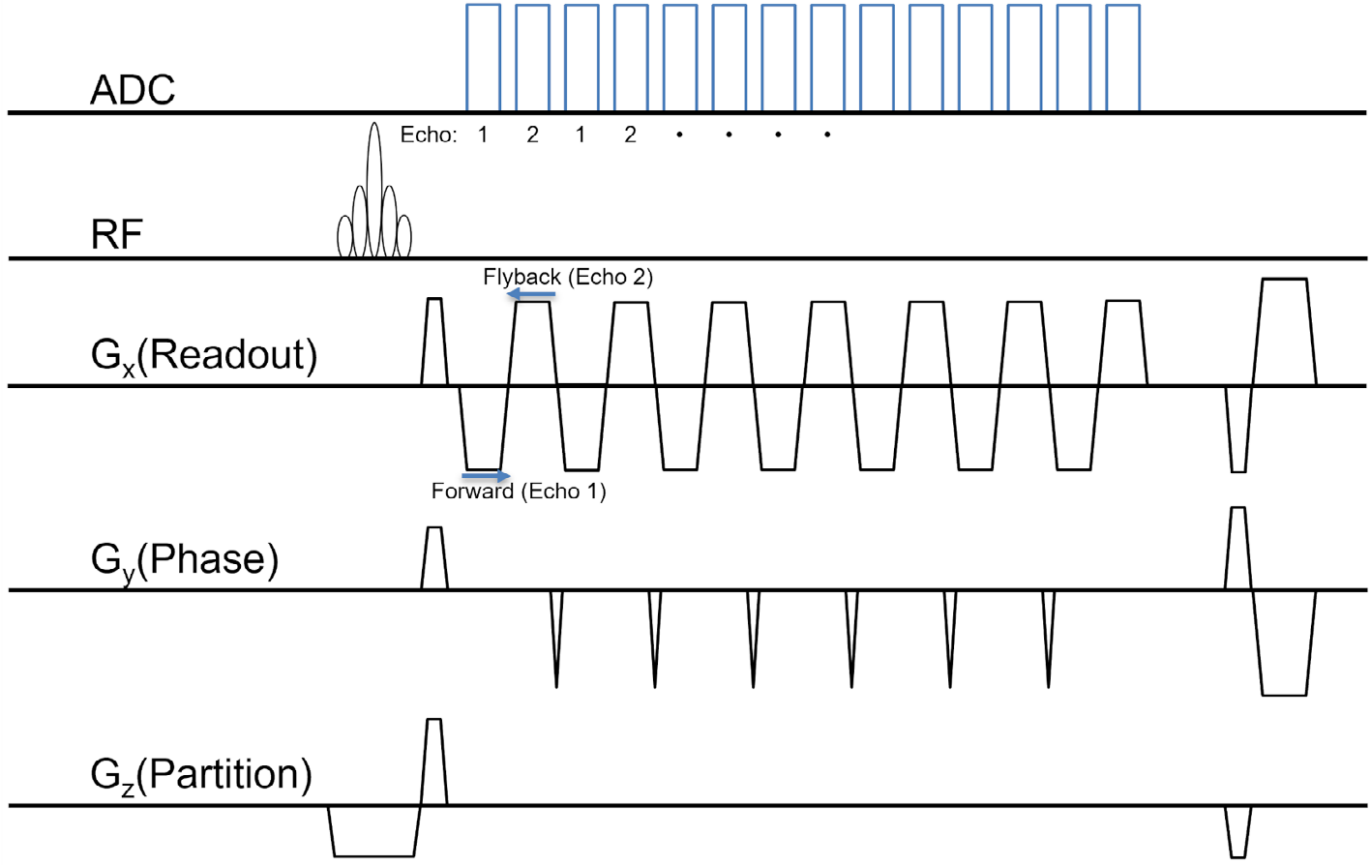
3D MR thermometry imaging (MRTI) pulse sequence implemented using a 3D segmented echo planar imaging (EPI) readout with dual‐echo acquisition (Echo 1 and Echo 2). Each k‐space segment is acquired with an oscillating readout gradient pattern, alternating between forward and flyback trajectories. This approach enables efficient sampling of two image contrasts within a single TR.

A 3D segEPI sequence with dual‐echo acquisition was implemented for volumetric MR thermometry, with attempts made to closely match the parameters for the previously described 2D MEMP sequence. This 3D sequence utilized two contrasts per TR, acquired using a forward readout followed by a flyback readout (see Figure 1). The imaging parameters were as follows: TR = 29 ms, TE = [13, 14.32] ms, FOV = [280 × 280 × 24] mm^3^, acquired matrix size = [256 × 133 × 8], spatial resolution = [1.09 × 2.19 × 3] mm^3^, flip angle = 12°, frequency encoding bandwidth = 930 Hz/pixel. Each imaging frame was acquired in approximately 3.3 s using an EPI factor of 7 and 6/8 partial Fourier sampling in the k_z_ direction (i.e., 6 of 8 k_z_ partitions were acquired and the remaining two were zero‐filled to reconstruct the full 3D volume). The two contrasts (echoes) were acquired by sampling the same k_y_ line in both directions—first with a forward readout and then immediately with a flyback readout before proceeding to the next k_y_ encoding step. This back‐to‐back acquisition resulted in a minimum echo spacing of 1.32 ms between the two TEs. The resulting 3D segEPI MRTI data was reconstructed using both individual and combined echoes. Both 2D and 3D temperature maps were echo‐combined without regard to the readout gradient direction since the magnitude of the expected heating‐induced off‐resonance shift in the hotspot focus in one direction (based on the amount of heating and readout bandwidth) was ≈0.06 mm for the 2D sequence and < 0.02 mm for the 3D sequence. Further, no opposing‐polarity shift in the hotspot was expected in the phase encoding direction between echoes for either sequence since all echoes acquired the phase encoding dimension of k‐space along the same direction.

### 2.1 Experimental Implementation Into Clinical Workflow

The 3D segEPI sequence was integrated directly into the existing clinical software environment, which connects the MRI scanner to the HIFU system via a vendor‐provided interface (AccessI, Siemens Healthineers). To maintain a clinically viable workflow without requiring modification to the HIFU software, additional functors were implemented within the MRI scanner's reconstruction frame (Image Calculation Environment, ICE, Siemens Healthineers). These functors enabled reconstruction of the full 3D dataset while passing only a single slice of the resulting volume to the HIFU software for real‐time MRTI processing and visualization. In this way, the volumetric sequence could be used clinically without changes to the clinical protocol, but the volumetric data could be retrospectively analyzed offline. Once regulatory approval and software updates to visualize volumetric data are available, it would be straight forward to send all reconstructed slices to the HIFU software for visualization and analysis.

For the comparison study, before each heating run, the sequence to be tested was named appropriately to be selected by the Insightec treatment software. Timing parameters were chosen to approximately match the acquisition time per dynamic frame between sequences. The acquisition time per frame entered into the clinical software needed to be rounded to the nearest tenth of a second for the user interface.

### 2.2 Experiments

#### 2.2.1 Phantom Experiments

To evaluate the accuracy and precision of measured temperature changes, the 3D segEPI and 2D MEMP sequences were performed on a tissue mimicking gel phantom (DQA phantom, Insightec) during focused ultrasound sonications. Ultrasound was delivered using the ∼1000 element clinical transducer operating at 650 kHz, with an acoustic power of 15.3 W for approximately 19.6 s. For each sonication, the measured temperature change, position of the focus, and the full width at half maximum (FWHM) of the heating profile were measured via 1D cross sections through the hotspot in each orthogonal direction. Heating experiments were performed 10 times successively (5 heatings for 2D MEMP, 5 heatings for 3D segEPI), alternating sequences for each sonication (i.e., 2D–3D–2D–3D–2D– …). Four to five minutes of cooling were allowed between sonications to allow the phantom to return to the baseline temperature.

#### 2.2.2 In Vivo Experiments

In vivo accuracy and precision in the clinical HIFU system was evaluated by performing 2‐min long scans in a healthy volunteer after informed consent using both sequences. Accuracy was defined as the root mean squared error of the median temperature value of the region over time, and precision was defined by the median of the temperature measurements' temporal standard deviation over the region of interest. These were calculated before and after phase drift correction [19] both over the whole brain region and over an 8 mm radius thalamic ROI, which is adequate to cover the target for clinical tremor (VIM ∼4 × 4 × 6 mm) using HIFU [20, 21, 22].

### 2.3 Image Reconstruction

To enable thorough analysis and comparison of the two acquisitions, the 2D MEMP and 3D segEPI image sets were reconstructed using Matlab (R2022b, MathWorks, Natick, MA, USA) code which mirrored the scanner reconstruction pipeline, employing EPI phase correction [23] and phase drift correction [19] before Fourier transformation and zero‐filled interpolation (ZFI). The segEPI sequence acquired a 3D slab consisting of an even number of slices (odd numbers of slices were not permitted by the pulse sequence) and was centered on the same location as the 2D slice. Hence, none of the slices in the 3D volume exactly overlapped the 2D slice position (i.e., the position of the two middle slices in the 3D volume “straddled” the position of the 2D slice). However, the position of the three‐dimensional voxel grid is arbitrary [24], and so can be adjusted to match the 2D position via a linear phase shift across the slice direction of k‐space so long as the volume of the 2D slice is contained within the excited 3D volume. Thus, to allow for accurate comparison, the 3D segEPI data in the offline reconstruction was shifted by ½ a voxel in the slice direction by applying a −π/2 to π/2 linear phase across k‐space in the k_z_ direction.

After appropriate ZFI and shifting of the voxel grid, PRF temperature change data was obtained via phase subtraction according to the following formula:

(1) $$\Delta T=\frac{\Delta \phi }{\gamma \alpha B_{0}\mathrm{TE}}$$

where ΔT is the change in temperature between frames, Δϕ is the change in image phase between frames, and α is the PRF temperature change coefficient (assumed to be −0.01 ppm/°C in this work). Phase differences for timepoint *n* were calculated as *arg* $\left(S_{n}S_{n-1}^{*}\right)$, where *S* is a complex image frame and $S^{*}$ is its complex conjugate. The final phase difference at each frame was then obtained as the cumulative sum of all previous phase differences to avoid potential phase wrapping. Combined‐echo PRF maps in both the 2D and 3D datasets were created by performing a weighted linear combination of the PRF maps from individual echoes, with the weightings given by ${\left(\left|m\right|\cdot \mathrm{TE}\right)}^{2}$ [9], where m is the complex image value (before PRF map creation) for each given voxel. After PRF temperature maps were created, the position of the hottest voxel was determined from the average of all five repeats of each acquisition protocol. Subsequently, the temperature at that voxel was plotted through time. To account for the slight difference in acquisition time and enable an accurate comparison, the 2D temperature curves were shifted in time by 0.42 s to produce maximal overlap between the time curves of the 2D vs. 3D sequence, as determined from a sum of squared error minimization. We hypothesize that this error is related to one of more discrepancies between the expected and actual trigger timing, which could be influenced by some combination of (1) the slight difference in acquisition time per frame, which could accrue across all the baseline acquisitions, (2) differences in reconstruction, processing, and/or data‐sending time between the MRI sequence and HIFU system for the two sequences that may have associated delays on the HIFU trigger. After the curves were time‐aligned, the 2D temperature curves were then temporally interpolated onto the timepoints of the 3D segEPI data (as opposed to the 3D data onto the 2D timepoints) since the 3D timepoints were found to straddle the peak heating time, and thus interpolation onto the 2D timepoints would produce artificially larger errors at the peak heating time. Bland Altman plots were used to determine the limits of temperature agreement, with an independent samples *t*‐test used to assess statistical significance.

## 3 Results

**FIGURE 2:**
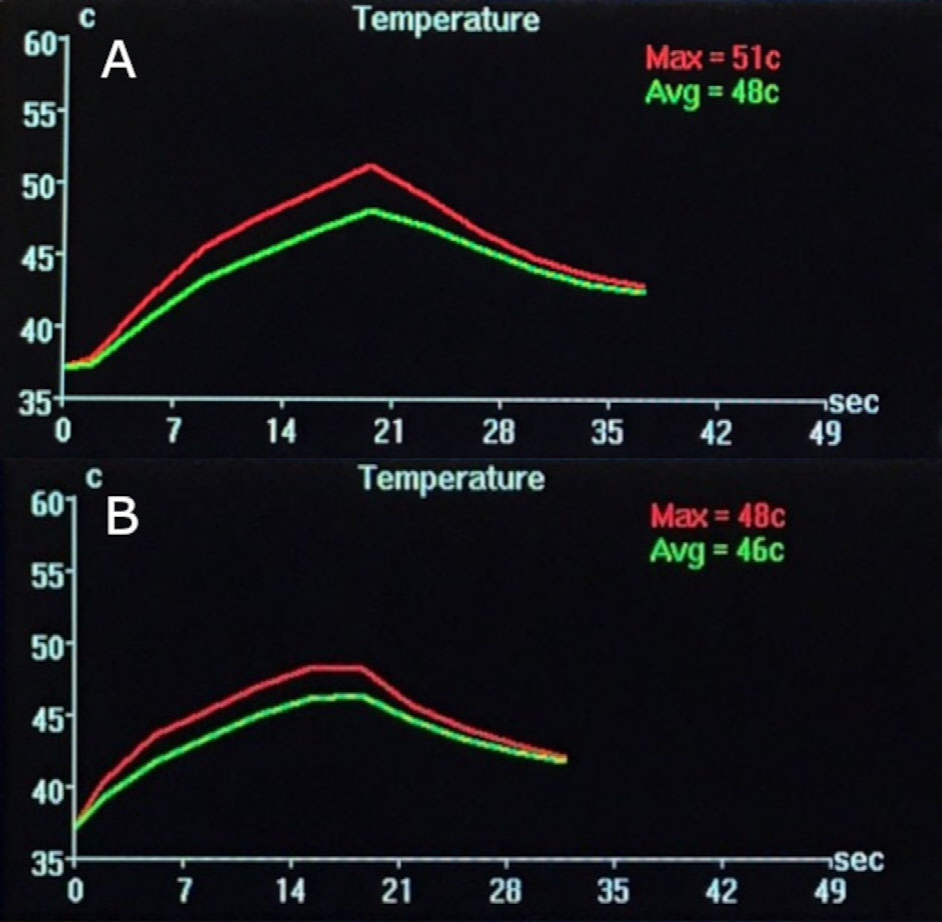
Representative temperature traces through time produced by HIFU software for 2D MEMP (A), and 3D segEPI (B).

Figure 2 shows representative online reconstructed temperature traces through time for the 2D MEMP (top) and 3D segEPI (bottom) sequences, as reconstructed by the Insightec HIFU system software, which indicates that 3D segEPI reconstruction can be integrated into the existing software successfully with a central slice passed for MR temperature calculation. Despite efforts to give approximately equal temporal footprint to the two sequences, there is a noticeable time shift in the reconstructed temperature curves, as especially evident in the starting heating time differences. This indicates that timing errors may be due to integration into the HIFU software. Additionally, shifting of the slab by half a voxel and ZFI were not performed in the online reconstruction, and time points were not co‐registered with each other.

**FIGURE 3:**
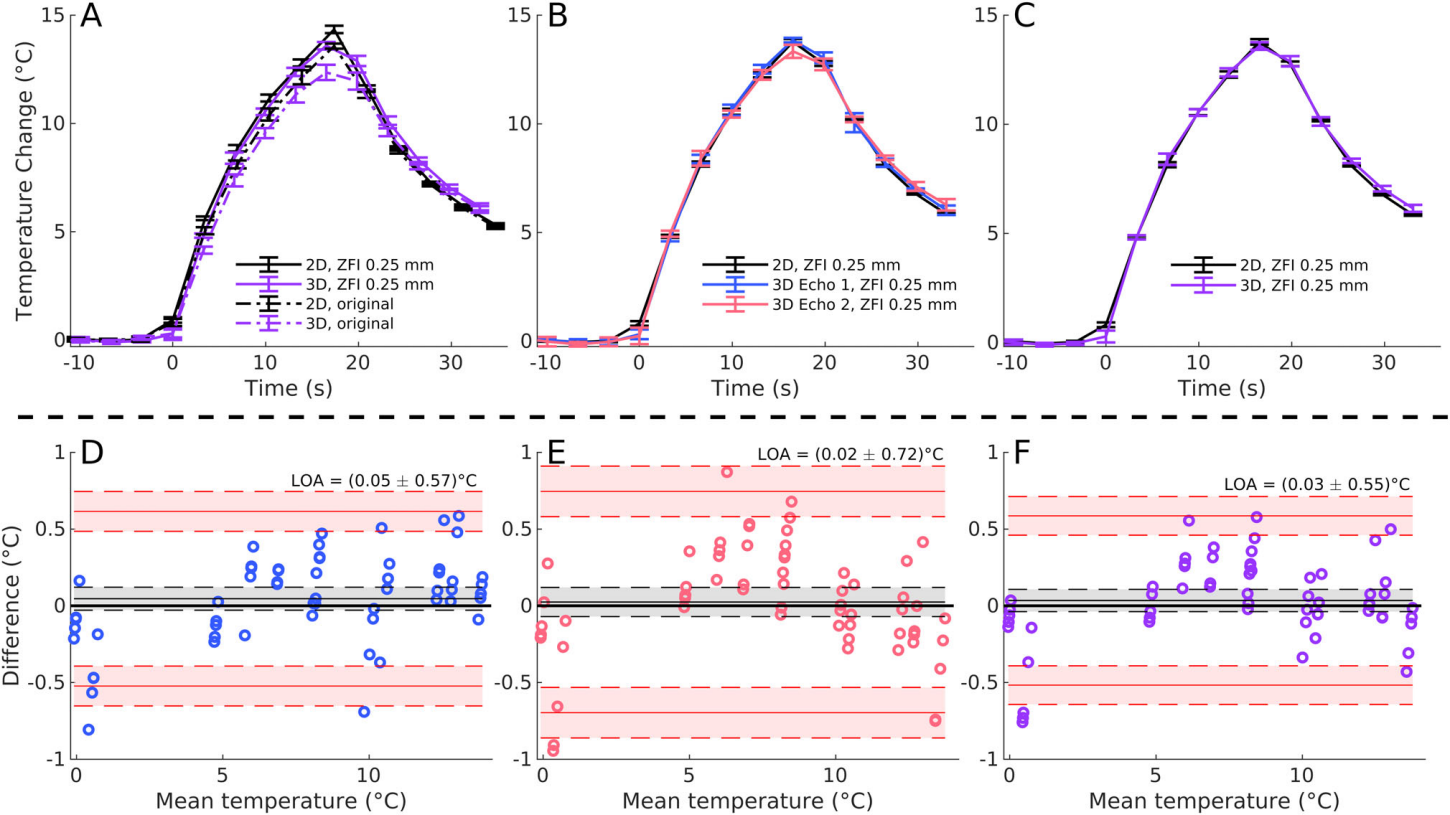
Hottest voxel comparison through time between 2D MEMP and 3D segEPI using the first echo (blue), second echo (red), and combined echoes (purple). (A–C) Temperature traces through the hottest voxel of each image set both before zero‐filled interpolation (ZFI, dot‐dash lines), and after ZFI to 0.25 mm in‐plane resolution (solid lines). (A) Original temperature traces without alignment or interpolation. (B) 2D traces for individual echoes' data of segEPI dataset are shifted by 0.42 s for time alignment, and then 2D traces are temporally interpolated onto 3D segEPI's time points. (C) Same shift as in (B), with the combined‐echo dataset shown. (D–F) Bland–Altman plots of data from first segEPI echo, second segEPI echo, and combined echo datasets, respectively, including data starting one timepoint before heating begins in each case.

Figure 3 shows the temperature trace through time of the hottest voxel of the focused ultrasound heating as measured by both 2D MEMP and 3D segEPI, with the mean ± SD shown. Both sets of images are reconstructed with our software in MATLAB. Figure 3A shows the unshifted traces both before (dot dashed lines) and after (solid lines) ZFI, with improved agreement between the two methods evident after ZFI is performed to 0.25 mm in‐plane resolution. Figure 3B shows the results after performing the uniform shift of the 2D MEMP trace by 0.42 s and linearly interpolating the 2D MEMP datapoints onto the 3D segEPI timepoints, with the PRF temperature data shown for the uncombined echoes. Figure 3C shows the results with the same shift as in Figure 3B, except the combined‐echo dataset is shown. Figure 3D–F shows Bland–Altman plots [25, 26] comparing the two sequences with the interpolations performed in Figure 3B,C for the first segEPI echo, second segEPI echo, and combined‐echo datasets, respectively. Agreement between the two methods was strong in all cases, with the beginning of ultrasound heating contributing the greatest systematic error. Despite this error near the start of the sonications, accuracy was generally high, with the limits of agreement in the Bland–Altman plots being between ±0.5°C and ±1.0°C.

**FIGURE 4:**
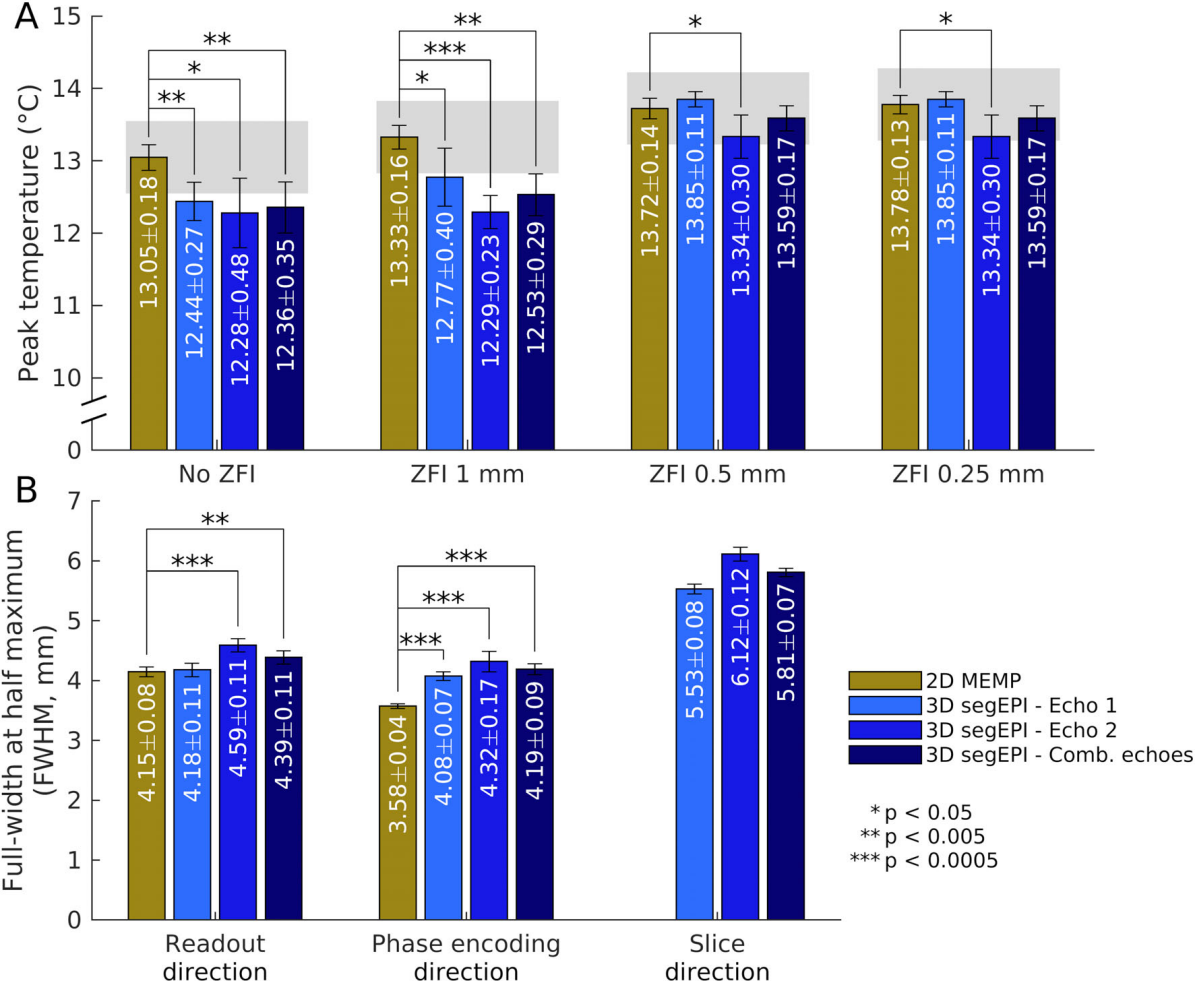
Comparison of 2D MEMP (gold) vs. 3D segEPI (blue) for phantom heating metrics for separate and combined echo datasets. (A) Comparison of peak temperature between sequences at the hottest voxel at various levels of ZFI. Substantially better agreement seen in cases of ZFI to 0.5 mm or finer in‐plane resolution. Gray shaded area shows 2D measurement ±0.5°C (B) Comparison of full‐width at half‐maximum along frequency‐encoding (readout), phase‐encoding, and slice‐encoding directions for dataset with ZFI to 0.25 mm in‐plane resolution. FWHM for the combined echo dataset is significantly larger in both readout and phase directions in segEPI, with a larger difference seen in the phase encoding direction. Asterisks indicate the level of significance.

Figure 4A shows the peak heating vs. degree of in‐plane ZFI, with ZFI not applied along the slice direction. Notably, the difference in peak temperature measurements from the interpolated 2D MEMP data (see Figure 3B,D) versus the 3D segEPI data at ZFI to 0.5 and 0.25 mm in‐plane resolution was not found to be statistically significant (*p* > 0.05). However, with decreasing levels of ZFI, the agreement between 2D MEMP and 3D segEPI diminishes, resulting in more substantial differences. Indeed, analysis of the agreement between varying levels of ZFI for a given sequence shows that ZFI produces notable and statistically significant temperature increases up to 0.5 mm (see Figure S1). After ZFI was done to at least 0.5 mm in‐plane resolution, the difference in peak heating between the 2D and 3D sequences was clinically negligible at < 0.5°C.

Figure 4B shows the full‐width at half‐maximum (FWHM) of the temperature change distribution for the 2D vs. 3D sequences in the three orthogonal directions as calculated from the images with ZFI performed to 0.25 mm isotropic resolution. For this work's implementation of 3D segEPI, the measured FWHM of the temperature distribution in 3D MRTI was systematically higher in both in‐plane directions, with the more substantial difference found in the phase encoding direction (∼17% larger). This is likely due at least in part to the smearing of the focus, which occurs when temperature‐induced off resonance causes signal shift along the low‐bandwidth phase encode direction to an increasing degree with increasing temperature [27]. The FWHM in the slice direction was longer than the in‐plane directions, as would be expected from a hemispherical transducer array. The cause of the smaller, but significant discrepancy in the FWHM along the readout direction is unknown. Interestingly, the second echo showed a systematically broader and lower amplitude heating distribution than the first echo.

**FIGURE 5:**
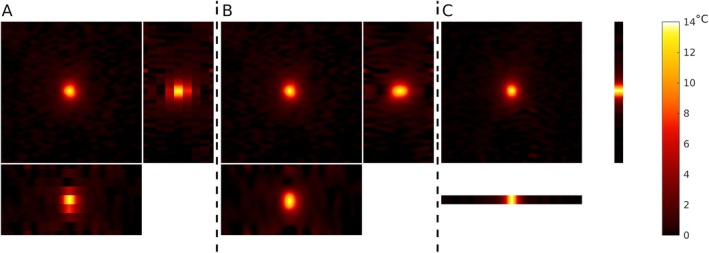
Hotspot heating comparison (°C) between 3D segEPI (A and B) and 2D MEMP (C) with orthogonal projections shown through the hotspot for 3D segEPI. (A) 3D segEPI heating with ZFI 0.25 mm in plane, but no ZFI performed in the slice direction. (B) Same as A, but with ZFI performed to 0.25 mm resolution in the slice direction. (C) 2D MEMP heating profile with ZFI 0.25 mm in plane. Note similarity in size, shape, and location of peaks between 2D and 3D, and the increased information about the extent of heating given in the 3D scan. The readout and phase‐encoding directions are vertical and horizontal, respectively, in the square images.

Figure 5 shows orthogonal views through the focal spot of a representative focused ultrasound heating from the phantom experiments. In Figure 5A, zero‐filled interpolation to 0.25 mm was performed only in‐plane, whereas the slice dimension was kept at 3 mm. Smoother looking temperature maps are clearly seen as ZFI is extended to the slice direction in Figure 5B. Clearly, the affected volume for a given sonication and the location of the focal heating in the slice direction can be more easily determined with a 3D sequence than with a 2D sequence.

Notably, the position of the focus in the 3D segEPI images was shifted back by around 2 voxels 0.5 mm (2 voxels in the dataset with ZFI to 0.25 mm in plane resolution) in the phase encoding direction relative to the focal position in the 2D MEMP images. With the given echo spacing of 2.64 ms, 6 partitions in the echo train, a peak heating around 13.7°C, and our field strength of ∼2.894 T, we would expect a shift of ∼0.27 voxels, which corresponds to just over 0.5 mm for the original 2 mm width voxels, which matches our observation. Additionally, the peak temperature position offset in the readout direction was [1, 0, 0, 1] voxels for the cases of no ZFI, ZFI 1 mm, ZFI 0.5 mm, and ZFI 0.25 mm, respectively. No additional shifting or distortion was expected in the second phase‐encoding direction of the 3D acquisition (the slice direction), since each slice was sampled in a separate TR with equivalent echo timing used across slices [28]. Correction of off‐resonance effects in reconstruction [27] could potentially remove the discrepancy in hotspot position between sequences.

**FIGURE 6:**
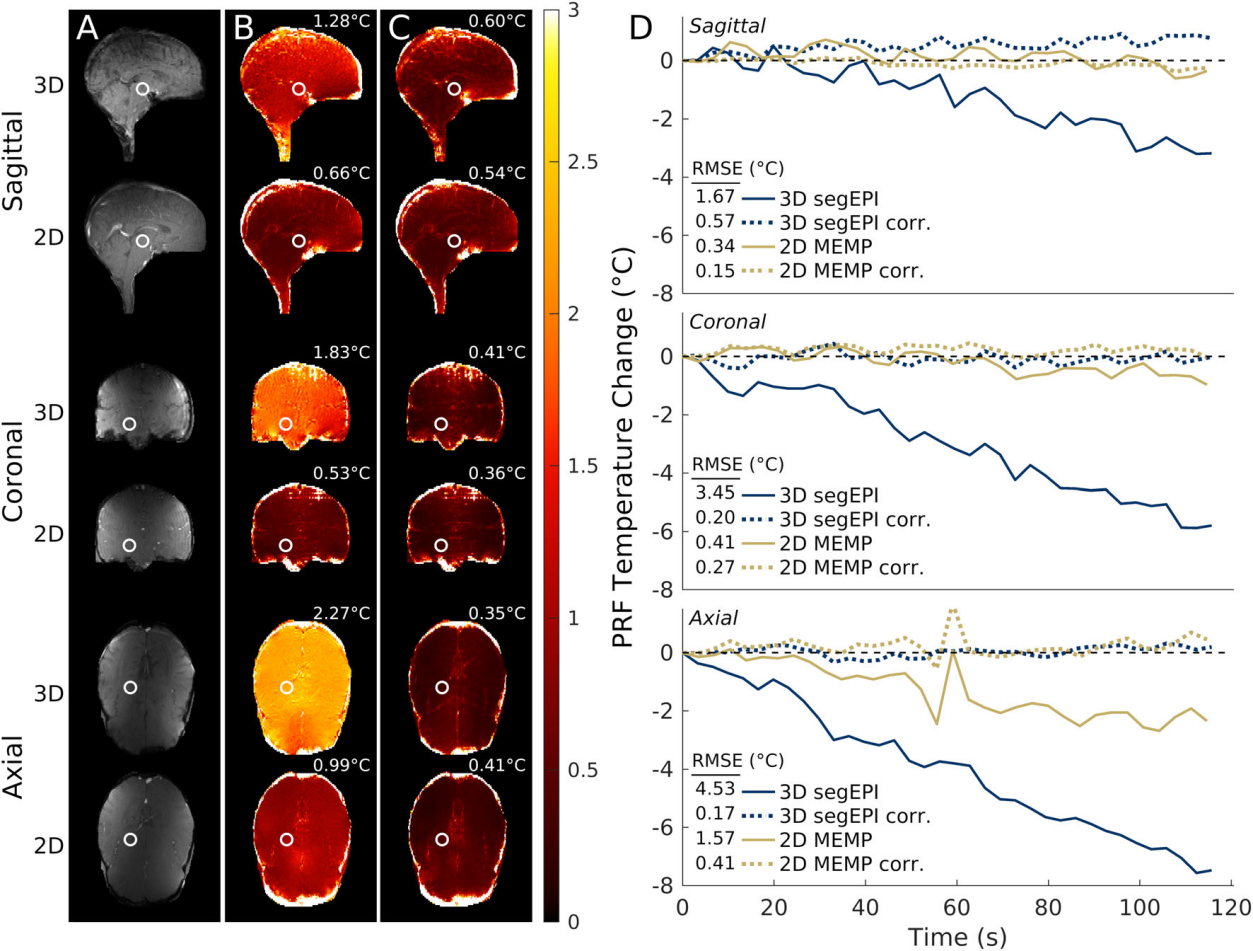
Precision of phase before and after phase drift correction of combined‐echo 3D segEPI and 2D MEMP in vivo brain images during 2‐min non‐heating experiments in the sagittal, coronal, and axial orientations. (A) Masked magnitude images obtained with 3D segEPI and 2D MEMP sequences inside Exablate transducer setup for each orientation. (B) Temporal standard deviation of the PRF temperature over the course of a 2‐min scan period without phase drift correction for each sequence and orientation. (C) Same as (B) with phase drift correction. (D) Average phase of the region‐of‐interest labeled with a white circle in each corresponding row of (A), (B), and (C) for the 3D segEPI vs. 2D MEMP sequences before and after phase correction. 3D segEPI phase drift is more substantial than that of 2D MEMP before correction in every orientation, but phase drift correction results in lower RMSE in all cases of both 2D MEMP and 3D segEPI. Central slice of 3D segEPI image set shown in A–C.

Figure 6 shows that phase drift correction, a critical step for long heating procedures, works suitably for both the validated 2D MEMP sequence and the 3D segEPI sequence. Before phase drift correction, the temporal standard deviation of the PRF temperature (Figure 6B) is relatively low (< 1°C) in all orientations for the 2D MEMP sequence but is substantially higher in all orientations for the 3D segEPI sequence, due to the pronounced, steady drift of the phase over time (Figure 6D, solid blue lines). However, after phase correction (Figure 6C,D, dotted lines), the phase remains stable over time without artefactual accumulation, and improvement is seen in all orientations for both 2D MEMP and 3D segEPI.

**FIGURE 7:**
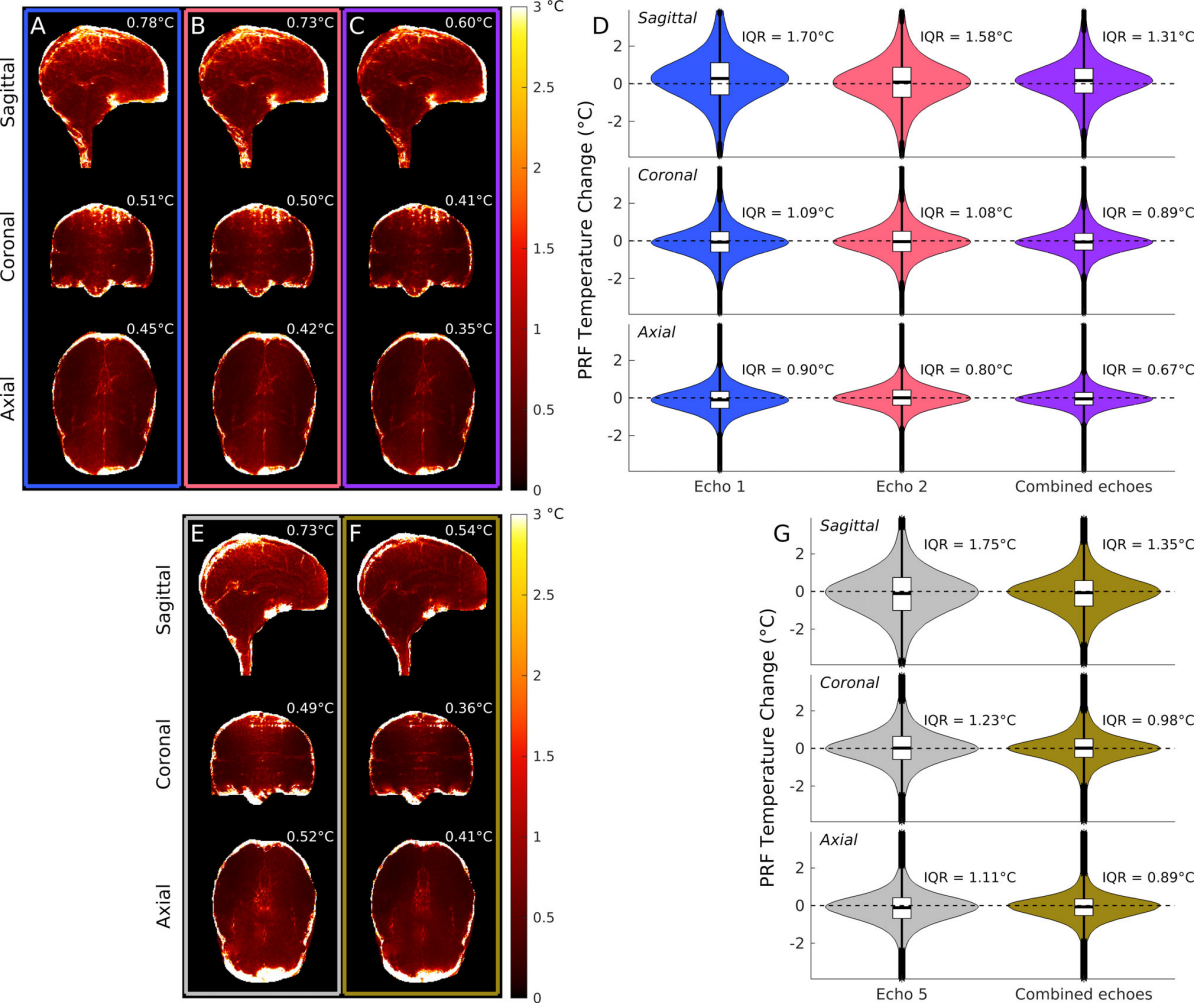
Comparison of PRF temperature precision through time in 2‐min non‐heating scans in sagittal, coronal, and axial orientations for 3D segEPI (A–D) using the first echo (blue), second echo (red), and combined echoes (purple) and for 2D MEMP (E–G) using the last echo (gray) and combined echoes (green). (A–C) Masked temporal standard deviation of the brain region across orientations from the 3D segEPI dataset with PRF temperatures produced using the first echo alone (A), the second echo alone (B), and both echoes combined (C). Value to the upper right of each image is the median of the temporal standard deviation across the masked voxels. (D) Violin plots of the distribution of all masked PRF temperature changes for the individual and combined‐echo datasets across the 2‐min non‐heating period at each orientation. Interquartile range for each orientation and dataset shown to the upper right of each violin plot. (E and F) Similar to (A–C), except the PRF temperature maps are created from the 2D MEMP using the last echo alone (E) and all five echoes combined (F). (G) Similar to (D), except the last echo and combined‐echo 2D MEMP datasets are used.

Figure 7 shows the impact of echo combination on PRF precision, obtained from the non‐heating dataset. In all orientations, precision improved upon combination of the first and second echoes of the segEPI dataset, as shown by the decrease in the median of the PRF temperature temporal standard deviation, as well as by the tightening of the interquartile range of PRF temperatures across brain voxels. Theoretically, the combination of multiple echoes with the weighting ${\left(\left|m\right|\cdot \mathrm{TE}\right)}^{2}$ mentioned previously should yield an improvement over the echo with maximal temperature precision. The degree of improvement, indicated with the ratio of the temperature standard deviation for the echo with the tightest precision, $\sigma_{\Delta T}^{\min}$, relative to that of the combined‐echo temperature map, $\sigma_{\Delta T_{\text{comb}}}$, is given by (see Appendix A for derivation)

(2) $$\begin{array}{c} \frac{\sigma_{\Delta T}^{\min}}{\sigma_{\Delta T_{\text{comb}}}}=\sqrt{\frac{\sum_{j=1}^{N_{\mathrm{eco}}}\left({\mathrm{TE}}_{j}^{2}\cdot e^{-\frac{2TE_{j}}{T_{2}^{*}}}\right)}{\max_{1\leq j\leq N_{\mathrm{eco}}}\left({\mathrm{TE}}_{j}^{2}\cdot e^{-\frac{2TE_{j}}{T_{2}^{*}}}\right)}}. \end{array}$$

Given a median T2 of ∼41 ms, as measured from the 2D multi‐echo acquisitions, the two 3D echoes at 13 and 14.32 ms respectively should yield a ∼37% increase in precision, whereas these results, when comparing the interquartile range or median temporal standard deviation of the individual echoes given in Figure 7 to the combined echo dataset, give an improvement of approximately 21%. Similarly, the combined echo 2D MEMP dataset showed only a ∼27%–33% improvement in precision compared to the last echo, which is a similar proportion below the maximal improvement of ∼61% for the combination of the five echoes' images.

## 4 Discussion and Conclusions

This work demonstrated that for the metrics of (1) accuracy of focal point location, (2) accuracy of focal point temperature, (3) extent of heating (FWHM), and (4) temporal stability, 3D segEPI can yield similar results to 2D MEMP with matched spatial and temporal acquisition parameters, provided sufficient ZFI is performed (to 0.5 mm in plane resolution) and proper consideration is made for the peak heating shift along the low‐bandwidth phase encoding direction. The limits of agreement for our implementation were better than ±1°C when ZFI to 0.5–0.25 mm in‐plane was done, with worse agreement seen at ZFI resolutions larger than 0.5 mm in‐plane. The additional 3D information provided by segEPI also allows for a more complete description of the extent of focused ultrasound heating than that provided by 2D MEMP. Though there were slight differences in the position of the focus (∼0.5 mm shift in phase encoding direction) and more prominent differences in the FWHM between the 2D and 3D sequences (also primarily along the phase encoding direction), these differences would likely be improved or resolved by correcting for temperature‐induced off‐resonance [27], which warrants further study. For comparison, ultrasound focal position adjustments in the clinic are typically on the order of 0.5–1 mm at a time, with new sonications and evaluation of treatment effect and side effects done in‐between each sonication and before any focal spot adjustment. It was further shown that high temporal stability of the phase is achieved in both 2D MEMP and 3D segEPI with use of Parker et al.'s phase drift correction algorithm [19], as employed in this work. Future work is needed to determine the cause of slight differences in the accuracy and larger differences seen in the FWHM that we observed between the uncombined echoes' datasets.

Additionally, this work demonstrates that a 3D EPI sequence can be successfully integrated into existing commercial HIFU software by sending a single reconstructed slice down the processing chain to replace the existing 2D slice. This relative ease of implementation could allow future pilot studies to acquire 3D temperature maps in vivo for retrospective analysis until the integration of the full 3D dataset can be approved by regulatory agencies and integrated into clinical software. This is a subject of future work. Further, while a dual‐echo segEPI sequence was implemented in this study, Figure 3 shows that the limits of agreement between a single echo segEPI (the version commonly available on most scanners) and 2D MEMP falls within ±< 1°C.

In our analysis, to achieve maximal alignment between the 2D MEMP and 3D segEPI temperature curves, a manual shift of 0.42 s was necessary. As seen from the temperature traces shown in Figure 2, there likely is a trigger timing discrepancy between the HIFU software and the MRI software that necessitated post‐processing alignment. This time discrepancy could be due to some combination of the slight acquisition time differences between the two sequences, processing time differences in the MRI reconstruction pipeline (e.g., performing two vs. three Fourier transforms, handling 8× the amount of data for the 3D case, etc.) and associated delays within the HIFU software, and trigger timing discrepancies. Improvements in the MRI and HIFU system's software handling of these differences should improve this discrepancy.

Interestingly, the agreement between 2D and 3D temperatures improved with further levels of ZFI, with the 3D temperatures showing higher sensitivity to ZFI. While the exact cause of this is unknown, one possibility is that the more pronounced heating‐induced off‐resonance shift of the 3D segEPI hotspot resulted in differing levels of temperature‐related partial volume effects between sequences in the larger, non‐interpolated voxels. Partial volume effects are known to effectively decrease with ZFI because it increases the uniformity of voxel sensitivity [24], and so the effect of different amounts of off‐voxel‐centered heating between the two sequences would be mitigated at higher levels of ZFI. Notably, Figure 4 shows that 0.5 mm × 0.5 mm in‐plane resolution was sufficient to bring the 2D and 3D temperatures into close agreement both in accuracy and precision, which agrees with the Todd et al.'s determination that ZFI to 0.5 mm in plane resolution was sufficient to produce high peak temperature precision [10].

Further, while the inclusion of an additional flyback echo in the 3D segEPI sequence did improve precision in the non‐heating scans, the improvement of 21% was less than the 37% expected. In the 2D case, the increase in precision over the latest echo (∼30%) was also around half that of the theoretical maximum improvement of 61%, as determined by Equation 2. This could be due to correlation in the background noise through time resulting from movement in the waterbath surrounding the skull, since the expected precision assumes that the noise across echoes through time is uncorrelated, as opposed to affected in part by a common time‐varying background.

Ultimately, this work shows the feasibility of replacing the 2D MEMP sequence with an equivalently timed 3D segEPI sequence for more complete monitoring of transcranial focused ultrasound heating procedures.

## Supporting information


**Figure S1:** Comparison of peak heating values obtained for 2D MEMP and 3D segEPI versus level of ZFI, using the same data as Figure 4. Peak temperature is found to increase significantly with increasing use of ZFI up to 0.5 mm in‐plane resolution. Gray shaded area shows 0.25 mm ZFI peak heating values ±0.5°C. Asterisks indicate the level of significance.

## Data Availability

The data that support the findings of this study are available from the corresponding author upon reasonable request.

## Appendix A

To derive the improvement in temperature precision obtained by echo combination, we begin with equations A6 and A8 of the derivation of the optimal weights for echo combination of multi‐echo PRF MRTI data given by Odéen and Parker [9], reproduced here.

(A1)
$$\begin{array}{c} {\sigma_{\Delta T_{j}}}^{2}=\frac{{\sigma_{m_{j}}}^{2}}{\gamma^{2}\alpha^{2}{B_{0}}^{2}T{E_{j}}^{2}{\left|m_{j}\right|}^{2}} \end{array}$$

(A2)
$$\begin{array}{c} {\sigma_{\bar{\Delta T}}}^{2}=\sum_{j=1}^{N_{e}}w_{j}{\sigma_{\Delta T_{j}}}^{2} \end{array}$$

Here, ${\sigma_{\Delta T_{j}}}^{2}$ is the variance of the temperature change ΔT measured for echo *j*, ${\sigma_{m_{j}}}^{2}$ is the variance of the complex image m at echo *j*, α is the PRFS temperature coefficient, ${\sigma_{\bar{\Delta T}}}^{2}$ is the variance of the combined‐echo temperature change, $N_{e}$ is the number of echoes, and $w_{j}$ is the weighting used for echo j in the echo combination.

Now we define the weighting factor to be proportional to ${\left(TE_{j}\cdot \left|m_{j}\right|\right)}^{2}$ as they suggest, such that

(A3)
$$\begin{array}{c} w_{j}=\frac{{\left(TE_{j}\cdot \left|m_{j}\right|\right)}^{2}}{\sum_{k=1}^{N_{e}}{\left(TE_{k}\cdot \left|m_{k}\right|\right)}^{2}} \end{array}$$

Then, we acknowledge that $\left|m_{j}\right|=Me^{-TE_{j}/{T_{2}}^{*}}$, and that all the terms in Equation A1 are constant across echoes except for the echo time and image magnitude, and thus can be denoted collectively as *C*. Then we combine Equations (A1), (A2), (A3) by taking the ratio of the minimum‐variance single‐echo temperature relative to the temperature variance of the combined echo dataset, and then taking the square root of that ratio. This is the relative improvement of the combined echo temperature precision over the best single‐echo temperature precision.

(A4)
$$\begin{array}{cc} \sqrt{\frac{\mathrm{mi}n_{j}\left({\sigma_{\Delta T_{j}}}^{2}\right)}{{\sigma_{\bar{\Delta T}}}^{2}}} & =\sqrt{\frac{\mathrm{mi}n_{j}\left(\frac{C}{T{E_{j}}^{2}\cdot {\left|m_{j}\right|}^{2}}\right)}{\sum_{j=1}^{N_{e}}\left[{\left(\frac{{\left(TE_{j}\cdot \left|m_{j}\right|\right)}^{2}}{\sum_{k=1}^{N_{e}}{\left(TE_{k}\cdot \left|m_{k}\right|\right)}^{2}}\right)}^{2}\frac{C}{T{E_{j}}^{2}\cdot {\left|m_{j}\right|}^{2}}\right]}}\\ & =\sqrt{\frac{\sum_{k=1}^{N_{e}}\left(T{E_{k}}^{2}\cdot e^{-\frac{2TE_{j}}{{T_{2}}^{*}}}\right)}{\mathrm{ma}x_{j}\left(T{E_{j}}^{2}\cdot e^{-\frac{2TE_{j}}{{T_{2}}^{*}}}\right)}} \end{array}$$

Notably, if we take the limiting case of zero time between echoes (i.e., $TE_{1}=TE_{2}=\mathrm{TE}$), then $\sqrt{\frac{\mathrm{mi}n_{j}\left({\sigma_{\Delta T_{j}}}^{2}\right)}{{\sigma_{\bar{\Delta T}}}^{2}}}=\sqrt{\frac{N_{e}{\mathrm{TE}}^{2}\cdot e^{-2\mathrm{TE}/{T_{2}}^{*}}}{{\mathrm{TE}}^{2}\cdot e^{-2\mathrm{TE}/{T_{2}}^{*}}}}=\sqrt{N_{e}}$, which is the well‐known expected precision improvement obtained from the average of $N_{e}$ equal‐variance estimates.
