# Supplementary material for: 3D MR Thermometry Using Bi‐Directional Segmented EPI for Transcranial‐Focused Ultrasound
Source: Magn Reson Med. 2025 Nov 23;95(4):2106–16. doi: 10.1002/mrm.70195 (PMC12850574; doi:10.1002/mrm.70195)
Supplement: Supplementary file 1 — Figure S1: Comparison of peak heating values obtained for 2D MEMP and 3D segEPI versus level of ZFI, using the same data as Figure 4. Peak temperature is found to increase significantly with increasing use of ZFI up to 0.5 mm in‐plane resolution. Gray shaded area shows 0.25 mm ZFI peak heating values ±0.5°C. Asterisks indicate the level of significance. [file MRM-95-2106-s001.docx]

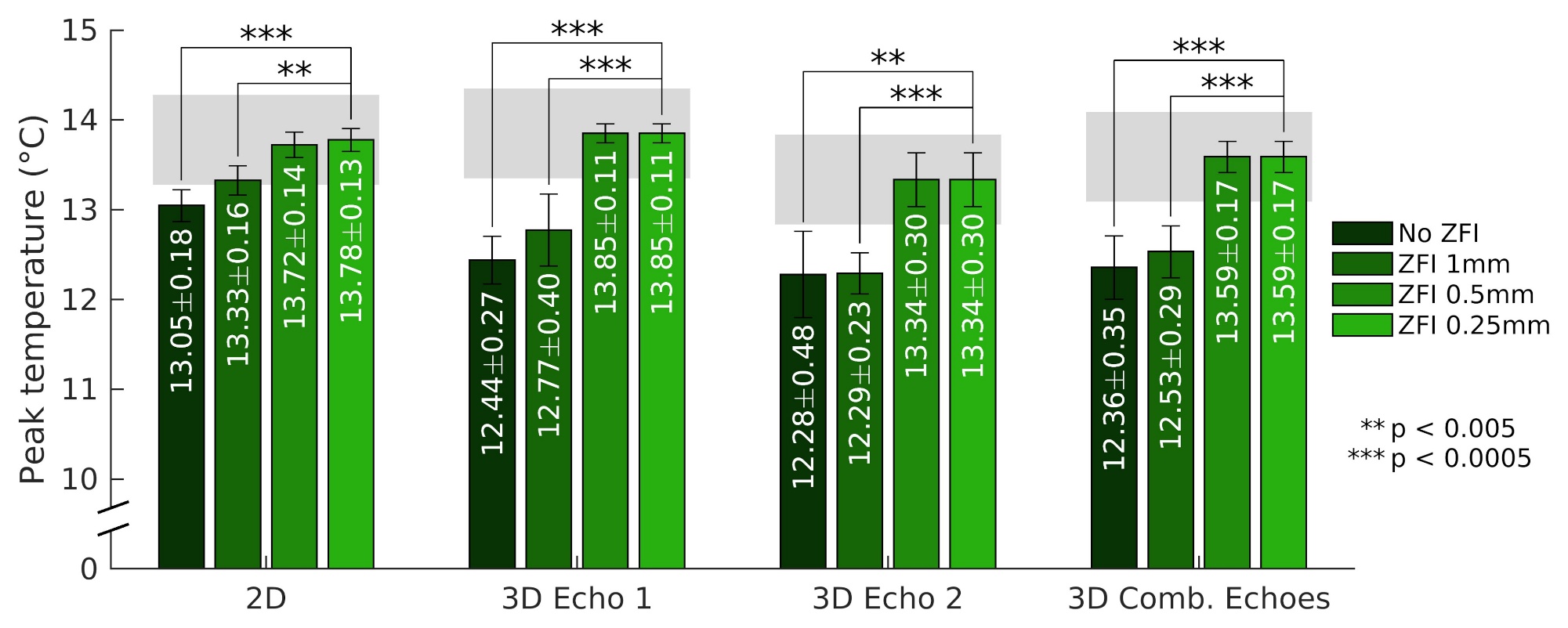


Figure S1: Comparison of peak heating values obtained for 2D MEMP and 3D segEPI versus level of ZFI, using the same data as Figure 4. Peak temperature is found to increase significantly with increasing use of ZFI up to 0.5 mm in-plane resolution. Gray shaded area shows 0.25mm ZFI peak heating values ± 0.5°C. Asterisks indicate the level of significance.
